# On Burns and Scalds

**Published:** 1807-09

**Authors:** 


### *To the Editors of the Medical and Physical Journal.*

GENTLEMEN,

THERE is no accident or disease, concerning the proper treatment of which there are more opinions, than in that of burns and scalds. Turpentine, cold water, chalk and vinegar, lime-water and oil, scraped potatoes, pounded onions, &c. &c. have been recommended by different practitioners, and every one seems to place as much confidence in the efficacy of his own remedy, as Mr. Perkins does in his metallic tractors; and, in my opinion, when any one remedy is used indiscriminately to every accident from fire, they have just as much reason to expect success.

If Drs. Kinglake and Kentish could be prevailed upon to join their plan of treatment, I am confident they would find their patients would be sooner relieved and cured, than by restricting themselves with empirical obstinacy to one favourite application.

Burns and scalds may, perhaps with propriety, be divided into three kinds, differing indeed only in extent.

The first division may include those cases, in which though the cuticle is raised in blisters, it still remains to cover the cutis.

The second those, in which the cuticle is stripped off, and the cutis exposed.

And the third, those in which the parts are so completely destroyed as to render a separation necessary.

In all the cases of the first description which I have met with, the application of cold water has been so universally successful, that I have never thought it necessary to seek for a more efficacious one. If an extremity has suffered, it may be plunged into a vessel of water, and kept there for any length of time with safety and advantage; age; indeed, so great is the mitigation of the pain, that the patient could not well be persuaded to remove it. If the injury has been inflicted upon the body, cloths wet with cold water, to which a small quantity of shrub has been added to increase the evaporation, will be found the best application; some caution will however be necessary, as inflammation of the viscera has sometimes been produced.

After a few days if the serum from the blister is not absorbed, it may be let out by *small* punctures, and the sores, if any exist, dressed with saturnine or calamine cerate.

The second division of cases are by far the most painful, and the most frequently fatal; few cases, if the injury is extreme, particularly in children, will do well under any mode of treatment. Symptoms of irritation come on immediately, and the patient frequently sinks in less than eight and forty hours. As death seems to be occasioned by the shock being greater than the constitution can recover, the indications of cure can never be to extinguish the small remains of life by the sedative effects of cold. And the cases of this kind, which I have seen, tend to support the opinion.

I have seen a child brought into a hospital, under the care of one of these extinguishing practitioners, and cooled to death's door, when the House Surgeon thinking the child would die, removed the cold applications, and gave the child some warm negus; it became much better and continued so till morning, when the cold was again applied, and with the same effect; till it was recovered by the use of cordials and warmth.

The plan I should recommend, would be to sheath the inflamed cutis as much as possible, and then lessen irritability, and support the patient with small quanties of wine. The first intention would be best answered by oil and lime water, cream, or milk and lime water. The second indication would be sought for in opium, which might be given in doses according to the pain, &c.

It is only in these cases, where there is destruction of parts, that I should ever think of using spirit of turpentine; in the first mentioned cases, it is unnecessary, and in the second it is, in my opinion, *highly improper*. Who would think of dressing a common blister, after the cuticle had been removed, with turpentine? What would be the consequence? Any man not biassed by prejudice, must know, that the most excruciating pain would be the actual consequence; and practice, (as far as my experience goes) confirms confirms the supposition. There may, perhaps, be some cases, in which, at the same time that the cuticle is repaired, the cutis may be so far destroyed, as to be immoveable to the stimulus of turpentine. In cases, where there are sloughs to be cast off, turpentine may hasten the separation, and it may lessen the fœtor from a large wound; this, in my opinion, is all a reasonable man can expect from it.

It may happen, that each of these cases may occur at the same time in the same patients. I should not hesitate to combine the methods of treatment I have mentioned, according as circumstances might require. I should never fear offending the constitution by different applications at one and the same time.

I am, &c.

A Member of the College.

*July* 9, 1807.

